# Evolution of Bipartite and Segmented Viruses from Monopartite Viruses

**DOI:** 10.3390/v15051135

**Published:** 2023-05-10

**Authors:** Hyunjin Park, Saven Denha, Paul G. Higgs

**Affiliations:** Department of Physics and Astronomy, McMaster University, 1280 Main St. West, Hamilton, ON L8M 4S1, Canada

**Keywords:** bipartite viruses, segmented viruses, assembly of viruses, evolutionary model

## Abstract

RNA viruses may be monopartite (all genes on one strand), multipartite (two or more strands packaged separately) or segmented (two or more strands packaged together). In this article, we consider competition between a complete monopartite virus, A, and two defective viruses, D and E, that have complementary genes. We use stochastic models that follow gene translation, RNA replication, virus assembly, and transmission between cells. D and E multiply faster than A when stored in the same host as A or when together in the same host, but they cannot multiply alone. D and E strands are packaged as separate particles unless a mechanism evolves that allows assembly of D + E segmented particles. We show that if defective viruses assemble rapidly into separate particles, the formation of segmented particles is selected against. In this case, D and E spread as parasites of A, and the bipartite D + E combination eliminates A if the transmissibility is high. Alternatively, if defective strands do not assemble rapidly into separate particles, then a mechanism for assembly of segmented particles is selected for. In this case, the segmented virus can eliminate A if transmissibility is high. Conditions of excess protein resources favor bipartite viruses, while conditions of excess RNA resources favor segmented viruses. We study the error threshold behavior that arises when deleterious mutations are introduced. Relative to bipartite and segmented viruses, deleterious mutations favor monopartite viruses. A monopartite virus can give rise to either a bipartite or a segmented virus, but it is unlikely that both will originate from the same virus.

## 1. Introduction

The simplest way of organizing the genes on a viral genome is via a monopartite virus in which all genes are linked in a single nucleic acid molecule. Usually, virus particles contain one copy of the viral genome per particle. In segmented viruses, genes are divided into two or more nucleic acid segments that are transmitted in the same particle. The virus particle then contains one copy of each of these segments. However, in bipartite and multipartite viruses, the genome is divided into two or more segments, and each of these is packaged into a separate virus particle. In order to infect a new cell, at least one virus particle of each type must enter the cell so that all the required genes of the virus are present.

The frequencies of viruses with these different types of genome organization differ between RNA and DNA viruses, between single- and double-stranded viruses, and among different types of host organisms [1]. It is estimated that a third of the viral genera infecting plants and fungi are multipartite [2]. These include plus-strand RNA viruses (e.g., Bymovirus and Benyvirus), minus-strand RNA viruses (e.g., Ophiovirus and Tenuivirus), double-stranded RNA viruses (Partitiviridae), and DNA viruses (e.g., Begomovirus and Nanovirus). RNA viruses also include families with segmented genomes (e.g., Bunyaviridae and Reoviridae). However, there are no examples of genera that contain both multipartite and segmented viruses. The widespread but patchy distribution of these virus types suggests that multipartite and segmented viruses have arisen independently several times, and that they can both originate from monopartite viruses. However, there may be factors that direct evolution towards either multipartite or segmented viruses in different cases rather than allowing both multipartite and segmented viruses to evolve from the same monopartite species. Among animal viruses, it is found that multipartite viruses are very rare, with the review of [2] citing only ssDNA bidensoviruses in silkworms and Guaico Culex virus, a ss(+)RNA virus, in mosquitoes.

Multipartite viruses are often seen as curious as the need for separate infection by more than one particle seems to be an obvious disadvantage [2,3]. Various possible advantages that might outweigh this have been proposed [4]. The most obvious of these is that shorter strands replicate more rapidly than longer ones inside a cell that contains both short and long strands. It is known that deletions occurring during the replication of a monopartite virus can produce shorter, defective genomes that lack some fraction of the genes necessary for the replication and transmission of the complete virus. These defective genomes can only multiply when present in the same cell as a complete virus. Defective viruses are commonly found in almost all types of viruses [5,6,7,8]. They are formed by deletions in complete viruses, a phenomenon occurring both in natural environments and in experimental situations where viruses are passaged on cell cultures. Defective viruses are known as defective interfering particles because they interfere with the replication and spread of the complete virus.

Two different defective viruses derived from the same complete virus will compete, and only the most rapidly multiplying defective will survive (unless there is continual rapid production of new types of defectives). The surviving defective will be the shortest sequence that still retains the ability to be replicated by the viral polymerase and to be packaged by the capsid proteins. However, if two defective viruses arise that encode complementary parts of the virus genome, they can multiply together when they co-infect a cell. At that point, they have the potential to operate together as a bipartite or segmented virus and, in some cases, they can eliminate the original monopartite virus. Populations with multiple types of defective viruses of different lengths have been seen in Flock House virus [7]. Specifically complementary pairs of defectives have been seen in the simian DNA birus SV40 [9] and the RNA virus foot-and-mouth disease virus [10].

This paper investigates the scenario in which two complementary defective strands arise from a monopartite virus, leading to the possibility of either a bipartite or a segmented virus. We aim to determine the conditions in which the bipartite or segmented form of virus outcompetes the monopartite form. There are several viral groups that fit this scenario of a bipartite virus originating from a monopartite virus very well. Within plus-strand RNA viruses, the genus Crinivirus is bipartite, possessing two RNAs of roughly equal length. Crinivirus is part of the family Closteroviridae [11], the other members of which are monopartite. Similarly, the genus Bymovirus is bipartite, while the other members of the Potyviridae are monopartite [12]. The Virgaviridae [13] contains the Tobamovirus, which is monopartite, and other groups (e.g., Tobravirus, Hordeivirus, etc.) which are bipartite; however, the ancestral state of this group is not clear. On the other hand, if the rooting of the phylogenies given by [14] are correct, the ancestral state of the family Secoviridae is bipartite. There are also some monopartite viruses within this group (Sequivirus and Waikavirus), which suggests that the fusion of separate strands can occur as well as the formation of shorter strands by deletion. However, whatever the direction of evolution, there is still a point at which there is competition between monopartite and bipartite viruses. Another relevant example is Ourmiavirus; this is tripartite and appears to be derived from monopartite viruses within the Botourmiaviridae [15]. An example from double-stranded RNA viruses is the bipartite group Partitivridae, which is related to monopartite groups Amalgaviridae and Hypoviridae [16].

The phylogenetic evidence for the origin of segmented viruses is less clear than that for bipartite viruses. Groups with segmented genomes often have multiple segments, implying that multiple events of segment splitting must have occurred, or that segments may have been gained from unrelated viruses by reassortment. One case where homology between segmented and monopartite viruses has been detected is that of Jingmen tick virus [17], for which two of its four segments show homology to monopartite Flavivirus genomes. Another case is Wenzhao tapeworm virus [18], which has two segments that are related to Nyamiviridae (a family of monopartite minus-strand RNA viruses). However, ViralZone [19] suggests that the apparent existence of two segments in this virus is an artifact of genome assembly. There is evidence that all minus-strand RNA viruses are monophyletic [16]. Many of these viruses are segmented, but there are also monopartite groups (Mononegavirales) which form a derived clade according to [16]. Thus, the common ancestor of minus-strand viruses may be segmented. Understanding relationships between segmented and monopartite viruses requires deep-level phylogenetics, the utilization of which is prone to uncertainties. Nevertheless, it is clear that the number of segments in segmented viruses can evolve and that the simultaneous origin of multiple segments seems unlikely; therefore, the scenario of competition between a monopartite virus and a two-segment segmented virus considered is relevant here.

We use stochastic models that describe the steps required for the multiplication of the virus inside one cell: the translation of the viral genes for the RNA-dependent RNA polymerase and capsid proteins, the binding of polymerases to RNA strands, the copying of a strand by a bound polymerase, and the assembly of virus particles. The advantage of a rapid replication rate applies both to short strands that are transmitted together as a segmented virus and to those that are transmitted separately as a bipartite virus. In this paper, we want to consider both the bipartite and segmented possibilities. Therefore, we need to consider the rates of assembly of particles containing different strands.

For some naturally occurring viruses, the assembly of virus particles containing RNAs of different sizes has been studied. Cowpea chlorotic mottle virus is a multipartite virus that forms three types of virus particles containing RNA strands close to 3000 nucleotides in length (nt). It is found that the same capsid proteins will encapsulate RNAs that can be several times longer than this [20]. The packaging of shorter strands of length 1500 nt or less is very inefficient. It has also been shown by computational modelling [21] that long RNA strands can steal capsid proteins from an equal mass of shorter strands, leading to faster assembly of particles with longer strands. If the capsid is icosahedral, then its shape and size will be determined primarily by the way the capsid proteins fit together. For this reason, the capsid may assemble less well when the size of the RNA does not match the natural size of the capsid. However, if the capsid is filamentous or rodlike, then the length of the capsid is determined primarily by the length of the RNA, in which case the assembly of the capsid may proceed efficiently irrespective of the length of the RNA. The ability of shorter RNA strands to spread as defective viruses or as components of a bipartite virus depends on their ability to be assembled. We therefore consider cases where the shorter strands assemble into particles as well as the full-length strands, and cases where they assemble less well.

For segmented viruses, the total amount of RNA in a virus particle containing one of each type of strand is the same as the amount in the full genome of the monopartite virus. Therefore, we might expect such particles to assemble efficiently because the capsid will still be of the correct size to fit the RNA. However, the formation of segmented particles requires the evolution of a mechanism of association of strands of the complementary types prior to the assembly of the capsid, or a mechanism of sequential addition of strands of the different types into partially formed capsids [22,23,24]. Such a mechanism is not necessary in a monopartite virus, and we assume that it does not exist initially at the time of origin of the separate defective strands. We suppose that an association mechanism subsequently arises that allows joint packaging, and we ask under what conditions such a mechanism is selectively favored.

It is difficult to experimentally study the competition between monopartite and bipartite viruses because most viruses exist as only one type or the other. However, a case of foot-and mouth-disease virus has been studied in which evolution from a monopartite to a bipartite virus has been found in the laboratory [10]. In this case, it was found that replication of the incomplete strands was not significantly faster than that of the complete strands. The advantage of the bipartite form was that virus particles containing defective strands were more stable, and hence more easily transmitted, than particles containing the monopartite genome. We note, however, that the deletions are very short in this example. The lengths of the defective strands are more than 90% of the full genome length, and several of the viral genes remain on both the defectives. If the length difference is small, this might explain why the more rapid replication of the shorter strands is not observed. This does not seem like a typical scenario for the evolution of bipartite viruses in nature, in which case the separate strands would be roughly half the length of the full genome assuming there were no genes present on either strand. If defective viruses formed initially by small deletions, these strands would not last long because further small deletions would occur until there were no remaining genes duplicated on either strand. Therefore, in our models, we assume the incomplete strands are considerably shorter than the complete virus, and that there is a definite rate advantage for the short strands.

The observation that particles containing the full genome are less stable than those containing slightly shorter defective viruses also seems unusual. This would imply that the original virus was not well adapted to its own capsid proteins. It should be easy for mutations in the capsid protein genes to adjust the shape and size of the capsid so that it is well matched to the monopartite genome. Thus, for most viruses, the stability of full virus particles should be greater than or equal to the stability of defective particles. We agree that if the bipartite particles were more stable, this would give a significant advantage to bipartite viruses. This case has already been studied in another theoretical model [25]. However, our models do not account for this effect because it does not seem to be a likely general feature. We assume that particles of all types are equally stable and transmitted equally well, but we do consider variations in the ability of strands of different lengths to assemble into particles, as described above.

## 2. Methods

We consider two different models for virus replication inside a cell, which we call the strand model and the assembly model. The strand model follows the steps of virus replication until the number of strands reaches a maximum $V_{max}$, at which point viruses are released from the cell. This model assumes that strands of all types are packaged equally well into virus particles and that each strand is transmitted in a separate virus particle. The assembly model includes the steps of virus particle assembly as well as replication and allows for the assembly rates of particles containing different strands to be different, and also for the assembly of segmented viruses with more than one strand per particle.

### 2.1. Strand Model

The model considers strands of three types: A, D and E. A is a complete monopartite virus that encodes all necessary genes. D and E are defective viruses that are complementary. Neither one alone encodes the full set of genes, but together they encode the full set. The two essential genes that will be included in the model are a polymerase gene and a capsid protein gene. A encodes both these genes. D encodes the capsid protein but does not encode polymerase, while E encodes the polymerase but does not encode the capsid protein. In an RNA virus, an RNA-dependent RNA polymerase must be encoded to allow for virus replication. In a DNA virus, an RNA-dependent RNA polymerase may not be necessary, but there will be other genes that are essential for replication, such as a virus-encoded protein that helps with the recruitment of host polymerases. It might be possible to refer more generally to a ‘gene essential for replication’, rather than a ‘polymerase gene’. However, we will use the term ‘polymerase’ in the text that follows for simplicity, and because our model assumes that polymerase proteins are translated from virus genes, and that these proteins then replicate the RNA strands.

Neither D nor E can replicate when alone, but both can replicate as defective viruses when in the same cell as A. As D and E have complementary genes, they can replicate when in the same cell and can survive as a bipartite virus in the absence of A for some parameter values. Let $V_{i}=W_{i}+X_{i}$, where $V_{i}$ is the total number of strands of type *i* = A, D or E, $W_{i}$ is the number of free strands (i.e., not bound to a polymerase), and $X_{i}$ is the number of strands that are bound to a polymerase. P is the number of free polymerases in the cell (i.e., polymerases that are not bound to a virus strand).

A cell is initiated by infection with a small number of strands of each type $n_{i}=0,1,2\ldots$ with a probability determined by the concentration of viruses released from previous generations of cells, as described in Section 2.3. Replication occurs via a series of stochastic events representing the polymerase binding, strand copying, and translation of polymerase genes. The rate of polymerase binding to type-*i* strands is $bPW_{i}$, where b is the rate constant for polymerase binding, a value which is the same for all strands. A binding event turns a free strand into a bound strand and reduces the number of free polymerases by one.

A strand copying event occurs at a rate of $kX_{i}L_{0}/L_{i}$. The rate constant for RNA polymerization, k, is the same per unit length for all template strands. Hence, the net rate for the whole strand is inversely proportional to its length. $L_{0}$ is the length of a complete viral genome in nucleotides. The length of the A strand is $L_{A}=L_{0}$, and the lengths of the defective strands are $L_{D}=L_{E}=L_{0}/2$. Hence, the rate of copying of the shorter strands is twice that of the full-length strand. A copying event releases the bound template strand as a free strand plus a free polymerase and creates a new free strand of the same type as the template. For simplicity, we do not distinguish between plus and minus strands of the virus.

The translation of polymerase genes occurs from A and E strands at a rate of $v(W_{A}+W_{E})$. A translation event produces one additional free polymerase. Polymerases are not translated from D strands because D strands lack the polymerase gene. This model does not consider the translation of capsid protein genes, but these are assumed to be present on A and D strands. Replication is only viable if the cell contains both capsid protein genes ($n_{A}+n_{D}>0$) and polymerase genes ($n_{A}+n_{E}>0$). When replication is not viable, any strands initially present in the cell are destroyed, and no viruses are produced.

In cells where replication is viable, the Gillespie algorithm [26] is used to simulate a stochastic series of events. The total rate of all events is $R_{tot}$. An event with rate $R_{event}$ is chosen with a probability of $R_{event}/R_{tot}$. The time is increased by an amount δt, which is a random variable chosen from an exponential distribution $p\left(\delta t\right)=R_{tot}exp(-R_{tot}\delta t)$. Replication continues until the total number of strands reaches the maximum allowed value, $V_{A}+V_{D}+V_{E}=V_{max}$, at which point virus particles are released containing one strand per particle.

### 2.2. Assembly Model

In addition to the steps in the strand model, the assembly model considers the steps related to capsid protein synthesis and virus particle assembly. The model tracks the number of capsid proteins in a cell, C. The number of capsid proteins required for a single virus particle, $n_{cap}$, may be quite large (e.g., $n_{cap}$ = 60 in an icosahedral particle of T1 symmetry, and 180 for T3 symmetry). For this reason, we measure C in units of $n_{cap}$ (i.e., C=1 represents sufficient capsid proteins to make one virus particle). Rather than allowing $n_{cap}$ individual translation events, which create one protein each (which would be very slow to simulate in the stochastic model), we consider a single translation event which create $n_{cap}$ proteins (C=1) in one step. Recalling that only A and D strands possess the capsid gene, the rate of this event is $v(W_{A}+W_{D})$, with the same rate constant v as for translation of the polymerase gene. This means that the translation of capsid genes is actually $n_{cap}$ times faster than the translation of polymerase genes. We assume that the virus is adapted for the rapid expression of capsid proteins relative to polymerases because it requires capsid proteins of order $n_{cap}$ per RNA strand but only of order 1 polymerase per RNA strand. If the virus were not adapted in this way, it would produce a great excess of polymerases.

In the assembly model, $Z_{i}$ denotes the number of assembled particles of type *i* in the cell. The assembly step converts a free strand into a virus particle by the addition of one unit of capsid proteins. This occurs at a rate of $a_{i}W_{i}{\left(C/C_{0}\right)}^{4}$. The rate of assembly is assumed to be linear in the number of RNA strands but non-linear in the number of capsid proteins—varying as ${\left(C/C_{0}\right)}^{4}$. The reason for this is that initiating a virus particle may require a nucleation step, involving the binding of several capsid proteins to an RNA strand simultaneously. $C_{0}$ is the capsid number per cell at which the assembly process begins to occur rapidly. The rate of virus assembly in the initial stages of the infection is very slow until C builds up to $C_{0}$. This allows the replication of a considerable number of virus strands to occur before encapsulation begins. The assembly rate constant $a_{i}$ may depend on the size and nature of the RNA that is encapsulated. The rate constant is set to $a_{A}=1$ for the A strand, while for the shorter defective strands, we set the rate to $a_{D}=a_{E}=a_{def}$. We consider cases where the defective strands assemble into particles equally well as the A strand ($a_{def}=1)$, and also where the defective strands assemble much less well ($a_{def}\ll 1).$

The assembly model also includes the possibility of forming segmented virus particles containing both D and E. We suppose that free D and E strands can associate to form a complex we denote as S for ‘segmented’. This occurs at a rate of $r_{S}W_{D}W_{E}$, where $r_{S}$ is the rate constant for association. The number of free S complexes is denoted as $W_{S}$. Complexes do not dissociate once formed, and polymerases cannot bind to these complexes, so that strands that form a complex cannot be replicated. Free S complexes $W_{S}$ are converted into complete segmented virus particles $Z_{S}$ at a rate of $a_{S}W_{s}{\left(C/C_{0}\right)}^{4}$. The assembly rate constant for the segmented particles is $a_{S}=1$, the same as for an A strand, because the segmented particle contains the same amount of RNA as the monopartite particle.

Rather than stopping replication when a fixed number of strands is reached, as in the strand model, the assembly model assumes that replication stops when the resources used by the virus for protein and RNA synthesis exceed the resources available in the cell. The number of protein resource units used by the virus is $PR=C+\sum_{i}Z_{i}$, which counts one unit for each unit of unassembled capsid proteins and one unit for each assembled virus. The resources used for polymerases are assumed to be negligible in this formula because they are less by a factor of $1/n_{cap}$. There is a maximum amount of protein resources in the cell ${PR}_{max}$. The translation of capsid and polymerase genes proceeds at the above rates as long as $PR<{PR}_{max}$. When PR reaches this limit, we set the translation rates to be zero, meaning that no further synthesis of viral proteins occurs.

The number of RNA resource units used by the virus is $RR=\sum_{i}\left(V_{i}+Z_{i}\right)L_{i}/L_{0}$, which counts one unit for each full-length strand synthesized, and half a unit for each defective strand of length $L_{0}/2$. The maximum quantity of available RNA resources in the cell is ${RR}_{max}$. RNA-binding and -copying steps proceed at the rates defined above as long as $RR<{RR}_{max}$. When this limit is reached, we set binding and copying rates to zero, meaning that no further RNA synthesis is possible. Polymerases already bound when this point is reached are assumed to detach from strands at a rate $10kX_{i}$ independently of the length of the strand. Detaching the polymerase releases the free template strand without creating a new strand. Free strands continue to assemble into virus particles after the cell resources are exceeded if capsid proteins are present in the cell. The process stops when a fixed time $t_{max}$ is reached. The number of virus particles of each kind released is the number $Z_{i}$ of assembled particles in the cell when this time is reached. We initially assume balanced resource conditions, where ${PR}_{max}={RR}_{max}=V_{max}$. This means that the cell has resources to make the same number of full-length RNAs as capsids. In this case, the number of viruses produced by the cell is always close to $V_{max}$, which is comparable to the value of $V_{max}$ viruses released by every cell in the strand model. Later, we also consider cases in which one of the resources is in excess relative to the other.

### 2.3. Transmission of Viruses between Cells

We consider a population of *N* host cells with separate host cell generations. The probability that a cell is infected by $n_{i}$ viruses of type *i* is a Poisson distribution

(1) $$P\left(n_{i}\right)=\frac{\lambda_{i}^{n_{i}}}{n_{i}!}exp(-\lambda_{i})$$

where the mean number, $\lambda_{i}$ is proportional to the number of viruses output by cells at the previous generation. In the strand model, this is

(2) $$\lambda_{i}=\alpha \frac{\langle V_{i}^{out}\rangle }{V_{max}}$$

where the triangular brackets denote the average over cells in the previous generation. The transmissibility, α, is a constant of order 1 that determines the rate at which the virus particles enter new cells. We have scaled the output numbers by a factor of $V_{max}$ because we expect the multiplicity of infection to be of order 1 while the number of viruses released per cell is large. With this scaling, a single type of A virus that produces $V_{max}$ viruses per infected cell will spread in the population if α>1.

In the assembly model, the assembled particles are transmitted; therefore,

(3) $$\lambda_{i}=\alpha \frac{\langle Z_{i}^{out}\rangle }{V_{max}}$$

We include the same scaling by $V_{max}$ in Equation (3) as Equation (2), so that the two models are comparable.

## 3. Results

### 3.1. Origin of Bipartite Viruses in the Strand Model

The following standard values of parameters are used in all results with the strand model. We set b=k=v=1, so that binding, copying and translation all occur on similar time scales. The time units used are arbitrary because only relative rates influence the numbers of different strand types produced. The number of viruses produced per infected cell is $V_{max}=100$. The length of the full virus $L_{A}=L_{0}=4000$, and the length of the defective strands is $L_{D}=L_{E}=L_{0}/2$, but only relative lengths influence the outcome.

Firstly, we consider the mean number of strands produced in one cell from the given initial numbers of infecting strands, as shown in Table 1. The mean output numbers are determined from an average of N=10,000 cells, all beginning from the same initial number of strands. When only A is present, $V_{A}^{out}=100$ exactly. For other combinations, the total of A, D and E is 100. When A is present with either D or E, it is significantly suppressed. It can be seen in Table 1 that E suppresses A more strongly than D does, even though D and E have the same length. It can also be seen that when D and E are in the same cell (either with or without A), more E than D is produced. E strands (which produce polymerases) have a slight advantage over D strands (that produce capsid proteins) in this model.

The difference between D and E is a somewhat surprising feature of the stochastic model. This difference does not arise in a deterministic model in which differential equations are written down for the mean numbers of strands per cell because the equations for the rates of change of D and E are the same if they have the same length. We attribute the difference observed between D and E in the stochastic model to the fact that the number of polymerases present in a cell is correlated with the number of polymerase genes, and hence the rate of replication is correlated with the number of E strands. In cells in which E strands are frequent, there are more polymerases produced and replication is faster, amplifying the number of Es. In cells in which D is frequent, there are fewer polymerases, and the replication of the D strands is slower. The net result is a higher average number of Es when averaged over many cells.

**Figure 1:**
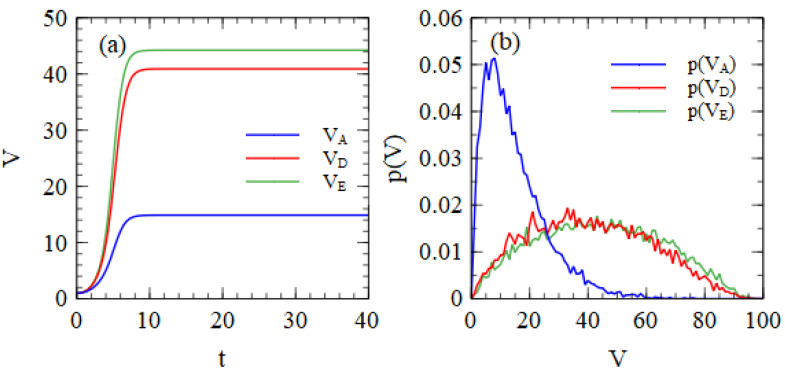
(**a**) Mean number of strands per cell as a function of time, beginning from one copy each of A, D and E. A denotes a complete virus; D is a defective strand encoding a capsid protein gene; and E is a defective strand encoding a polymerase gene. (**b**) Probability distribution of the number of output viruses of types A, D and E, beginning from one copy of each.

The numbers in Table 1 are the mean numbers of viruses produced per cell. There can be substantial variation from one cell to another, even when cells begin with the same number of strands in the initial infection. Figure 1a shows the mean numbers of strands of A, D and E per cell as a function of time, beginning from one copy of each. Replication in each cell is stopped when the total number of strands reaches 100. The time units are arbitrary, but all the cells have reached 100 strands by the time *t* = 40. Figure 1b shows the probability distribution of the number of output strands after all strands have reached 100 total strands. These distributions are very broad.

**Figure 2:**
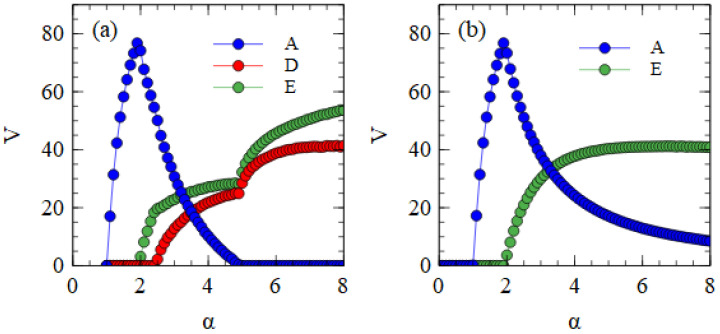
Mean numbers of viruses produced per cell as a function of transmissibility α, which determines the mean number of viruses that infect a single cell (as in Equation (2)). (**a**) When D and E are complementary, the combination of D + E eliminates A at high α. (**b**) When D and E are not complementary, E survives as a parasite of A, and D is eliminated.

We now consider the transmission of strands over multiple cell generations. The population size is *N* = 10,000. We begin with $\lambda_{i}$ = 1 for each of A, D and E, and determine λ in subsequent generations from the previous output numbers, as in Equations (1) and (2). Note that each cell in a generation has different numbers $n_{i}$, determined independently from the same Poisson distributions, and that the values of $\lambda_{i}$ are different for each type of strand because the output numbers of strands are different. The transmissibility α is the same for each kind of strand. Figure 2a shows the mean output numbers of viruses per cell in the steady state as a function of α. Separate simulations are performed for each value of α, and the virus numbers are averaged over cells and over cell generations once steady-state numbers are reached.

Several regimes are visible in Figure 2a. For α ≤ 1, all types of strands die out because they are not transmitted with sufficient frequency. For α > 1, the complete monopartite A virus can survive alone. As α increases, the fraction of cells infected by A increases and eventually becomes high enough to support the transmission of D and E as defective viruses. Since E suppresses A more strongly than D (as shown in Table 1), E can survive with A at a lower value of α than D. In the range 1.9<α<2.5, only A and E survive. For α>2.5 all three strands survive. As α increases further, D and E suppress A to a greater extent until a point is reached where A is driven to extinction. For α>5.0, D and E survive as a bipartite virus in the absence of A.

The extinction of A only occurs because D and E have complementary genes. To demonstrate this, in Figure 2b, we consider the case where D and E are not complementary. In this case, when D and E are in a cell with A, the model is the same as before, but when D and E are in the same cell without A, no replication occurs and there is no output of viruses from this cell. These simulations begin with A, D and E all present, but D is eliminated in all cases. Therefore, there is no curve for D in Figure 2b. When the two defectives do not have complementary functions, E eliminates D because it is a better parasite of A. E is dependent on A; therefore, it cannot cause the extinction of A.

The strand model gives a simple explanation of why bipartite viruses arise. Some authors have looked for reasons as to why using separate particles might be advantageous. We suggest that the only advantage of D and E is that they are shorter and replicate faster than A when in the same cell. There is no advantage to being in separate particles. The two parts are packaged in separate particles by default because they are both derived from defective viruses of the same monopartite virus. The monopartite virus packages one complete strand per capsid; hence, it is likely that there should be one defective strand per capsid unless some new mechanism evolves that causes packaging of two strands in the same capsid (thus forming a segmented virus). Our model shows that the bipartite virus sometimes outcompetes the monopartite virus at sufficiently high α without the need to evolve a mechanism of segmented particle assembly.

The strand model does not yet consider the possibility of segmented viruses. In order to do this, it is necessary to allow for some possibility of packaging D and E in the same particle. Hence, we need a model that includes steps related to capsid production and assembly. The assumption of a fixed number $V_{max}$ of virus particles produced per cell seems too simple when we consider segmented viruses. Is the number of virus particles produced limited by the number of RNA strands or the number of capsids? This distinction does not matter if there is one strand per capsid because the number of strands is equal to the number of capsids. However, this is not true if we allow more than one strand per capsid. For these reasons, we find that the strand model is not sufficient to consider the origin of segmented viruses. As such, we now turn to the assembly model, which is able to address these issues.

### 3.2. Origin of Bipartite and Segmented Viruses in the Assembly Model

The following standard values of parameters are used in all results with the assembly model. We set b=k=v=1, $L_{A}=L_{0}=4000$, and $L_{D}=L_{E}=L_{0}/2$, as in the strand model. The assembly rate for particles containing A strands is always $a_{A}=1$. The assembly rate for defective strands D and E is $a_{def}$, and we begin with the case where this is also 1. The capsid number at which assembly becomes rapid is $C_{0}=20$. We begin with the case where there is no association between D and E, ($r_{S}=0).$ A cell has a limiting amount of protein resources, ${PR}_{max}$, measured in units of number of virus capsids that can be synthesized, and a limiting amount of RNA resources, ${RR}_{max}$, measured in units of the number of full-length RNA strands that can be synthesized. Initially we set both limits to be equal, ${PR}_{max}={RR}_{max}=V_{max}$, with $V_{max}=100$. We refer to the case where both limits are equal as ‘balanced resources’ (BR).

**Figure 3:**
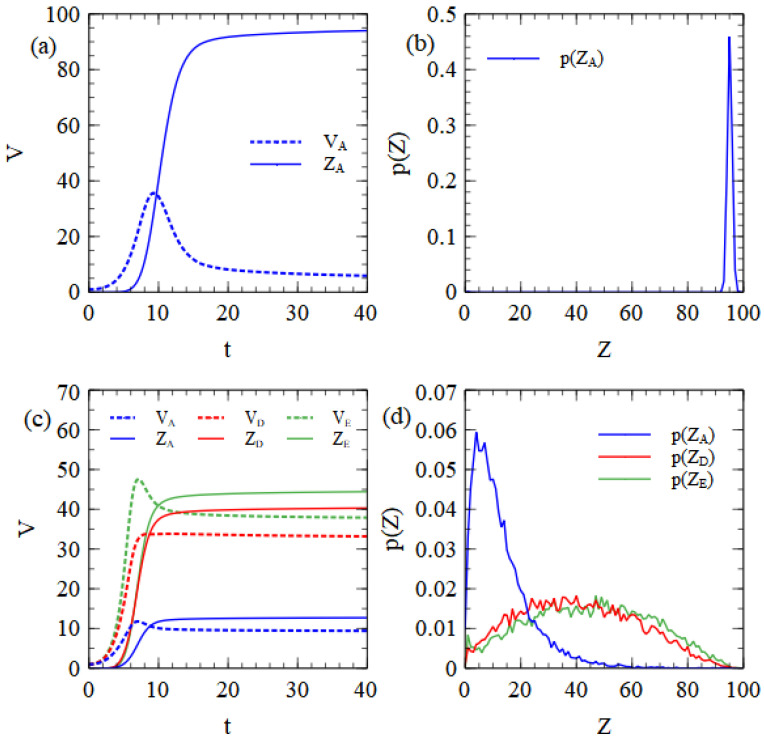
Results from the assembly model with balanced resources. (**a**) Mean numbers of unpackaged virus strands, $V_{A},$ and complete virus particles, $Z_{A}$, per cell as a function of time, beginning from $n_{A}=1$. (**b**) Probability distribution of the number of complete virus particles produced per cell beginning from $n_{A}=1$. (**c**) Mean numbers of unpackaged strands and complete virus particles of three types, beginning from $n_{A}=n_{D}=n_{E}=1$. (**d**) Probability distribution of the number of complete virus particles produced of three types, beginning from $n_{A}=n_{D}=n_{E}=1$.

Figure 3a shows the mean number of unpackaged strands, $V_{A}$, and complete virus particles, $Z_{A}$, per cell as functions of time. Unpackaged strands initially increase, but then decrease once packaging becomes faster than copying of new strands. After the resources are used, there is no further synthesis of capsids or RNA strands and the remaining capsids and strands are slowly assembled into virus particles. As the number of remaining capsids becomes low, the assembly rate drops greatly because it depends on ${\left(\frac{C}{C_{0}}\right)}^{4}$. A small number of strands therefore remains unpackaged when the maximum time $t_{max}=40$ is reached. This seems to us to be a reasonable feature of the model that is likely to be true in real viruses.

We observed that if the fourth-power dependence is replaced by a linear dependence on C, there are fewer leftover unpackaged strands with long times, but that virus assembly is too rapid in the early stages of infection. There is a possibility that all virus strands become encapsulated when they are still few in number and that this prevents further replication before the resources of the cell are exhausted. This latter result does not seem realistic; therefore, we stick to the fourth-power dependence. The mean number of complete particles produced is 94.1 in this example. The probability distribution of the number of particles produced per cell (see Figure 3b) is a sharp peak close to the mean. The monopartite virus is well adapted to these balanced resource conditions, since in most cells it uses both protein and RNA resources to the limit and manages to produce a number of particles that is very close to the limit given by the cell resources.

Figure 3c shows the mean numbers of unpackaged strands and virus particles as a function of time, beginning from a single strand of each of A, D and E. In this case, the RNA resource limit is 100, but the D and E strands are half-length, and only count for half a resource unit. Up to 200 D and E strands can be synthesized, which is a quantity larger than the limit on the capsids (which is still 100). A substantial number of unpackaged strands therefore remain in the cell when all the capsid proteins are turned into virus particles. The distribution of the number of particles produced of each type is shown in Figure 3d, and this is very similar to the corresponding results for the strand model shown in Figure 1b.

The mean numbers of virus particles produced from different combinations of starting strands are shown in Table 2. The top five lines consider cases where $r_{S}=0$, meaning that no S complexes can be formed. These results are very similar to those obtained for the strand model in Table 1, with the exception that the total number of particles produced is slightly less than 100 instead of exactly 100. We refer to the case where $a_{def}=1$ and $r_{S}=0$ as fully bipartite since only separate D and E particles are produced. The bottom five lines in Table 2 consider cases where $r_{S}>0$ and segmented particles can also form. In all cases, the assembly rate constant for segmented particles is $a_{S}=1$, the same as is obtained for monopartite A particles. We refer to the case with $a_{def}=1$ and $r_{S}=0.01$ as mostly bipartite. With this slow rate of complex formation and fast rate of assembly of defective particles, most of the D and E strands end up in separate particles and few segmented particles are produced.

We attempted to increase the number of segmented particles by increasing the rate of association between D and E to $r_{S}=0.1$. However, unexpectedly, this leads to a slight reduction in the number of S particles and to a considerable reduction in the number of D and E particles. The problem is that D and E now form the complex S rapidly, which allows for rapid packaging into S particles but stops the replication of D and E. Additionally, the number of copies of D and E in a cell is not always balanced and, if the last copy of the rarer strand forms a complex, then no further replication of that strand is possible. Thus, the rapid formation of the S complex does not favor the production of more S particles. Increasing $r_{S}$ above 0.1 leads to the production of even fewer S particles.

In contrast, the number of S particles can be increased substantially if the rate of assembly of the single D and E particles is reduced to $a_{def}=0.01$, while $a_{S}=1$ and the rate of formation of the complex remains low at $r_{S}=0.01$. We refer to this parameter combination as mostly segmented because a large number of S particles is now produced, while there are fewer D and E particles. If $a_{def}=0$, we have obtained the fully segmented case in which only S particles are produced and D and E strands cannot form separate particles.

The mean number of S particles in the fully segmented case is only 51.8. This value is much lower than the maximum of 100 that can be obtained from the available resources, and much lower than the number of monopartite viruses produced (94.1) when the monopartite virus is alone in the cell. The reason for this is that, even if a cell begins with $n_{D}=n_{E}=1$, and even if the two strands replicate at the same average rate, the numbers of copies of the two strands do not remain equal. After one replication, we have a 2:1 ratio; if the next strand to be replicated is chosen randomly from the three, we are twice as likely to go a 3:1 ratio than to go to 2:2. By the time large numbers of strands are copied, it is likely that one of them is significantly more frequent than the other. If the RNA resource limit is 100, the total number of half-length strands produced is 200, meaning that there is an average number of 100 copies of each of D and E produced per cell. However, the rarer of the two strands is likely to have significantly fewer than 100 copies. In the fully segmented case, the maximum number of S particles possible is equal to the number of copies of the rarer of the two strands, which is usually much less than 100. This significant disadvantage of the segmented virus with respect to the monopartite virus arises from the stochastic replication process in the model, and this seems to be a realistic feature that will be experienced by real segmented viruses. It is not easy to see how a real virus could manage to achieve more balanced numbers of copies of the two strands than is created by random replication. The problem is significant if the D and E strands have equal length, as considered here, and it would be even worse if the length of the strands were unequal since the longer strand would usually be much rarer.

The final line of Table 2 shows that when A, D and E are all in the same cell, more S particles are produced than A particles. For this reason, it is still possible for a segmented virus to outcompete a monopartite virus despite the fact that fewer S than A particles are produced when they are in separate cells, as we will now show.

**Figure 4:**
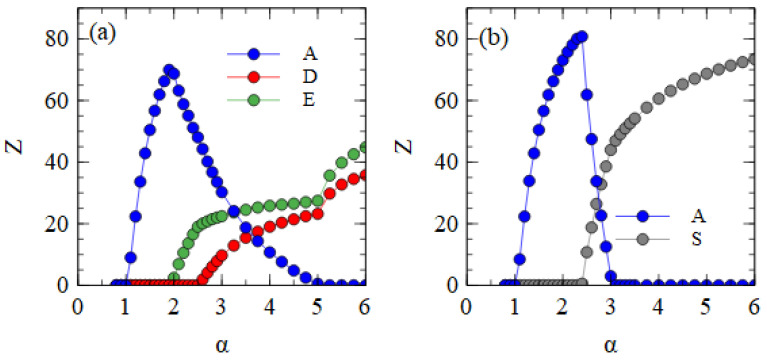
Mean numbers of virus particles per cell as a function of α in the assembly model with Balanced Resources. (**a**) Fully Bipartite case. (**b**) Fully Segmented case.

We now consider transmission between cells using the assembly model. Figure 4a shows the mean numbers of A, D and E particles produced per cell, averaged over time, in the fully bipartite case where $a_{def}=1$ and $r_{S}=0$. The bipartite case in the assembly model is very similar to the strand model (shown in Figure 2a). There is a regime with only A at low α; this is followed by regimes of A + E, and A + D + E as α increases; these are followed by a regime with only D and E.

Figure 4b shows the fully segmented case where $a_{def}=0$ and $r_{S}=0.01$. In this case, there is a regime of only A at low α, followed by a narrow regime where A and S coexist, followed by a regime at high α where the segmented virus eliminates the monopartite virus. This confirms that the advantage of S when in the same cell as A can outweigh the smaller production rate of S when they are in separate cells as long as transmissibility α is high enough.

### 3.3. Evolution of an Assembly Mechanism for the Segmented Virus

A comparison of the two cases in Figure 4a,b shows that the minimum α required for the bipartite virus to eliminate the monopartite is 5, while the minimum α required for a segmented virus to eliminate the monopartite is 3. This may suggest that the using the segmented virus is a “better” strategy than relying on the bipartite virus, and moreover seems to confirm our intuition that packaging the two strands in the same particle makes more sense than packaging them separately. However, we are assuming that the D and E strands are defectives that arise via deletions in the A virus. As the A strands assemble into particles containing a single strand, it is likely that the D and E strands will assemble separately into particles containing a single strand unless a mechanism evolves that causes the association of D and E prior to assembly into a virus particle.

In this section, we suppose that D and E strands originally form without the ability to associate ($r_{S}=0$), but that then a variant of D arises, which we call D*, that possesses some element of sequence or structure which allows it to associate with E. Both D and D* can form particles with a single strand, but only D* can form S particles. We wish to determine whether D* is selected relative to D.

**Figure 5:**
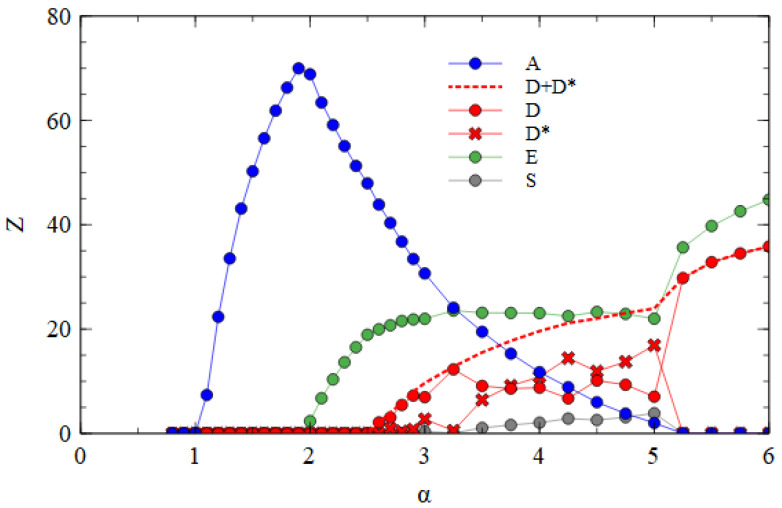
Mean numbers of virus particles per cell as a function of α in the mostly bipartite case.

Figure 5 shows the mostly bipartite case, with $a_{def}=1$ for all short strands, i.e., D, D* and E, and $r_{S}=0.01$ for the D* and E strands. As always, $a_{A}=a_{S}=1$. For each value of α, the simulation is initiated with A, D, D* and E strands present and allowed to proceed until a steady state is reached. E survives with A for α > 1.9. D and D* survive for α > 2.4. These limits are the same as those shown in Figure 4a, which only has one D variant. Small numbers of S particles are also formed once α is large enough for D* to survive. However, we have already seen in Table 2 that very few S particles form when $a_{def}=1$. This occurs because the D* strands are usually packaged separately before they can form the complex. Therefore, very few S particles are formed in the middle range of α in Figure 5.

Given that few S particles are formed, there is little difference between D and D*. The data points for D and D* fluctuate up and down rather randomly, whereas the total of D and D* (dashed line) follows a smooth curve. This is a sign that the amounts of D and D* fluctuate by random drift and that there is no indication of selection in favor of D*. For α > 5, the bipartite virus eliminates the monopartite virus. At this point, D* is also eliminated, leaving only D and E. This shows that in cases where the bipartite virus does well, there is selection against the D* variant that forms the association with E. Thus, in conditions where D and E strands assemble efficiently into separate D and E particles, a mechanism that leads to the formation of S particles is not selected by evolution.

**Figure 6:**
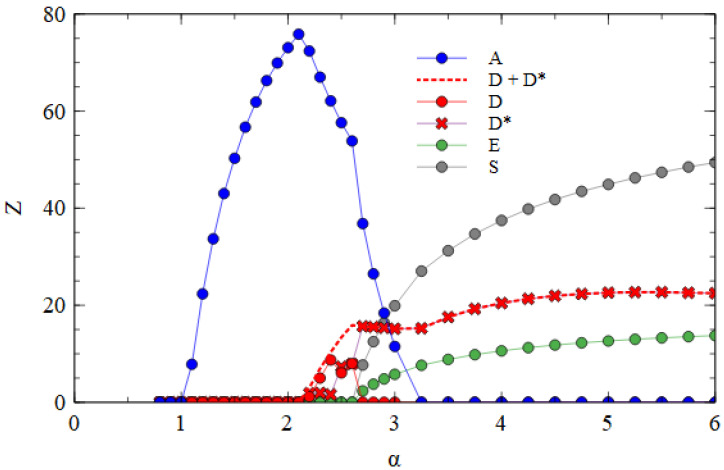
Mean numbers of virus particles per cell as a function of α in the mostly segmented case.

Figure 6 shows the mostly segmented case, where $a_{def}=0.01$ for all short strands D, D* and E, and $r_{S}=0.01$ for the D* and E strands. For these parameters, D survives at a lower α value than E. As D is the strand that produces capsid proteins, having a higher concentration of capsid proteins is an advantage when the packaging of defective strands is slow. The fact that E produces additional polymerases, which gives an advantage for E in previous cases, is less important when $a_{def}$ is low. In the range 2.2 ≤ α ≤ 2.6, D and D* survive with A. As there is no E in this range, there is no difference between D and D*, and the frequencies of D and D* can fluctuate due to random drift, with only the total of D and D* being under selection. For α > 2.6, E also survives. At this point, D* and E start to form S complexes and S particles. We see in Table 2 that substantial numbers of S particles form for these mostly segmented parameters. In Figure 6, the number of S particles rises quickly with α. As soon as E survives (α > 2.6), D* is selected and D is eliminated. For α ≥ 3.25, A is also eliminated, leaving only D* and E strands which are transmitted mostly as S particles with smaller numbers of D* and E particles. Thus, if defective strands are not packaged efficiently into separate particles, a mechanism that causes the association of D* and E into segmented particles is selected by evolution. The resulting segmented particles compete successfully against the original monopartite virus and sometimes eliminate it. We have supposed that it is the D strand that evolves the new variant D*. However, we could equally well have considered E evolving into a variant E*, or into two variants D* and E*. We expect all these cases to be very similar.

### 3.4. Effect of Varying Resources

So far, we have considered balanced resources (BR) where ${PR}_{max}={RR}_{max}=V_{max}$. Resource limits are a property of the host cell, and there is no reason why these should always be such that an equal number of capsids and RNA strands can be synthesized. Now, we consider the effect of varying the relative amounts of protein and RNA resources. We define excess protein resources (XPR) as cellular conditions where ${PR}_{max}>{RR}_{max}$. We simulate the case where ${PR}_{max}={3V}_{max}$, and ${RR}_{max}=V_{max}$, keeping $V_{max}=100.$ We define excess RNA resources (XRR) as cellular conditions where ${RR}_{max}>{PR}_{max}$. We simulate the case where ${RR}_{max}={3V}_{max}$, and ${PR}_{max}=V_{max}$, keeping $V_{max}=100.$ Table 3 shows mean numbers of viruses produced per cell under each resource condition for the monopartite, fully bipartite, and fully segmented viruses, scenarios in which each type of virus is alone in the cell.

The monopartite virus does well in BR conditions, producing 94.1 viruses, a number which approaches the maximum limit of 100. Table 3 shows that the numbers of monopartite viruses in the XPR and XRR conditions are 99.9 and 98.0, and these values are only slightly higher than for BR conditions. Increasing the levels of one resource over the other does not make much difference to the monopartite virus because it is still limited by whichever of the two resources is the lower.

The bipartite virus also does well in BR conditions, producing 97.2 particles in total. However, for BR, up to 200 half-length strands can be produced, and only half of these can be packaged. For XPR conditions, up to 300 units of capsid proteins can be produced, which is enough to package all the 200 strands that are produced. Thus, the number of bipartite virus particles produced is roughly doubled in XPR conditions, which is a significant advantage. On the other hand, for XRR conditions, up to 600 half-length strands can be produced; however the maximum number of particles is still limited to 100 by the capsid proteins. Therefore, increasing RNA resources makes little difference to the bipartite virus.

The segmented virus has a significant disadvantage in BR conditions because the two strands tend to be produced in unequal numbers and as the number of segmented particles produced is limited by the rarer of the two. Up to 100 units of capsid proteins can be synthesized, but only 51.8 S particles are formed on average, leaving many capsid proteins remaining that do not end up in virus particles. In XPR conditions, up to 300 units of capsid proteins can be synthesized, but this simply increases the number of excess capsid proteins that do not form particles. Therefore, increasing protein resources does not benefit segmented viruses. On the other hand, for XRR conditions, up to 600 half-length strands are produced, and the distribution of the numbers of strands for each type is broad over the range from 0 to 600, with the mean being close to 300. In this case, there are more copies of the rarer strand than for the BR case, and there is a good chance that even the rarer strand has more than 100 copies. The mean number of S particles formed increases to 68.3, which is a big improvement from 51.8. Thus, increasing RNA resources gives a significant advantage to segmented viruses.

**Figure 7:**
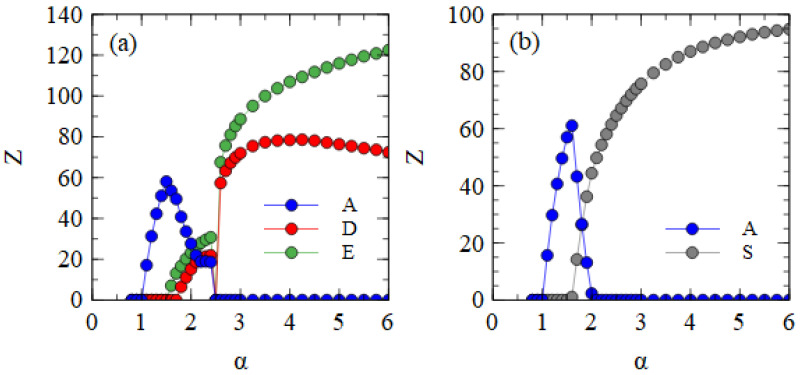
(**a**) The fully bipartite case with excess protein resources. (**b**) The fully segmented case with excess RNA resources.

Figure 7a shows the mean number of viruses produced per cell in the steady state in the fully bipartite case with XPR. As expected, XPR gives an advantage to bipartite viruses. The curves are shifted to the left relative to those shown for BR in Figure 4a. The minimum α at which the bipartite virus eliminates the monopartite is 2.5 for XPR conditions and 5.0 for BR conditions. With XRR conditions, there is no extra benefit provided to the bipartite virus. The minimum α at which the bipartite eliminates the monopartite remains at 5.0.

Figure 7b shows the mean number of viruses produced per cell in the fully segmented case with XRR. As expected, XRR gives an advantage to segmented viruses. The curves are shifted to the left relative to those obtained for BR shown in Figure 4b. The minimum α at which the segmented virus eliminates the monopartite is 2.0 for XRR conditions and 3.1 for BR conditions. With XPR conditions, there is no extra benefit provided to the segmented virus. The minimum α at which the segmented eliminates the monopartite is 3.0, almost the same as the value for BR.

### 3.5. Effect of Deleterious Mutations

RNA viruses are known to have low-fidelity polymerases with high mutation rates. We therefore investigate the effect of deleterious mutations on the competition between virus types. The bipartite case is well described by the simpler strand model; therefore, we return to the strand model to look at deleterious mutations. An A virus with working versions of both capsid and polymerase genes is denoted as $A_{cp}$. Deleterious mutations are denoted as 0. Strands with deleterious mutations in one or both genes are denoted as $A_{0p}$, $A_{c0}$ or $A_{00}$. $D_{c}$ is a strand with a working capsid gene. $D_{0}$ is a strand with a deleterious mutation. $E_{p}$ is a strand with a working polymerase gene. $E_{0}$ is a strand with a deleterious mutation. Strands with mutant genes produce no proteins from the mutant genes, but they can still be replicated and transmitted between cells. Each time a sequence is copied, there is the probability u of a deleterious mutation occurring in each gene, and a probability 1−u of correctly copying the gene. Thus, copying a $D_{c}$ gives a $D_{0}$ with probability of u. Copying an $E_{p}$ gives an $E_{0}$ with probability u. Copying an $A_{cp}$ gives an $A_{0p}$ or $A_{c0}$ with probability u(1−u) or an $A_{00}$ with probability $u^{2}$. The reverse mutation from a 0 to a functional gene is assumed to be negligible.

**Figure 8:**
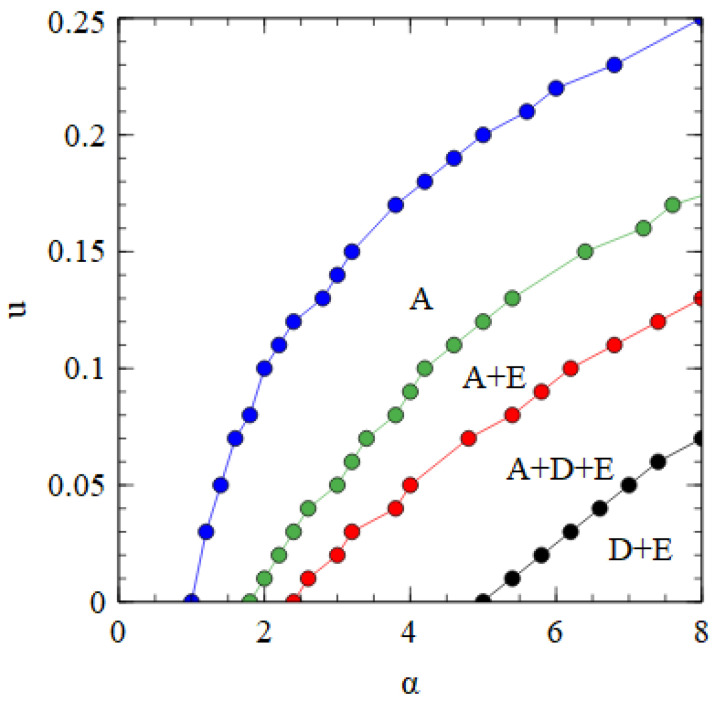
Phase diagram in α, u space for bipartite and monopartite viruses in the strand model. α is the transmissibility, and u is the probability of a deleterious mutation per gene replication event.

Figure 8 shows a phase diagram indicating which viruses survive in which regions of the α,u parameter space. The horizontal axis with u=0 is equivalent to Figure 2a. There is a region at high α and low u where only the bipartite virus D + E survives. By either decreasing α or increasing u, we move through regions of A + D + E, A + E, and only A, and eventually reach a region where no virus survives. The upper boundary line is the error threshold of the A virus. For mutation rates above the error threshold, the virus is eliminated by the accumulation of deleterious mutations.

**Figure 9:**
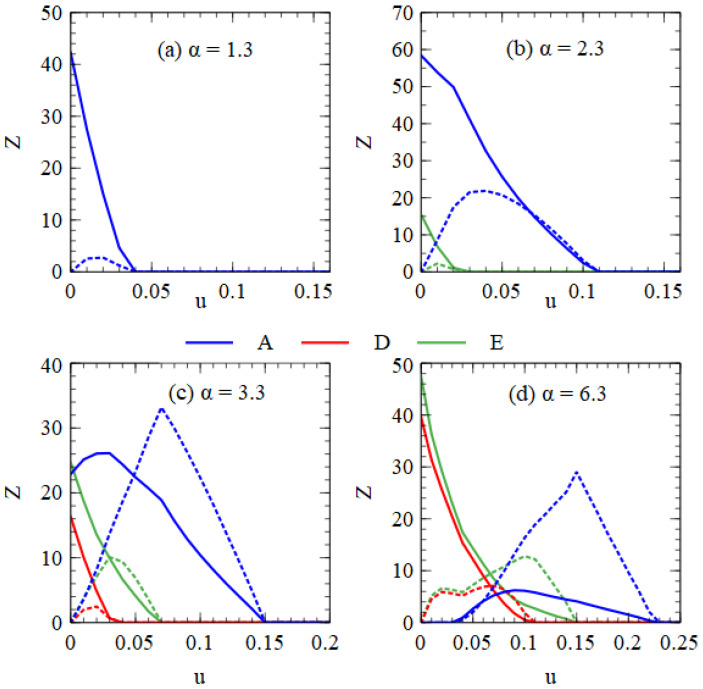
Error threshold curves showing virus numbers as a function of *u* for 4 fixed values of α. (**a**) α = 1.3, (**b**) α = 2.3, (**c**) α = 3.3, (**d**) α = 6.3. Functional A, D and E strands are labelled blue, red and green. Mutant strands are labelled with dotted lines of the same colour. The curve for the mutant A strands shows the sum of the numbers of the three types of A mutants.

Figure 9 shows error threshold curves obtained by plotting virus numbers as a function of *u* at fixed α values. In Figure 9a for α = 1.3, we are in the regime of only A when *u* = 0. As *u* increases, we reach the error threshold for A. In Figure 9b for α = 2.3, we are in the regime of A + E when *u* = 0. As *u* increases, we pass through the error thresholds for E and A. In Figure 9c for α = 3.3, we are in the regime of A + D + E when *u* = 0. As *u* increases, we pass through the error thresholds for D, E and A. In Figure 9d for α = 6.3, we are in the regime of D + E when *u* = 0. As *u* increases, we pass through the point where A first appears, followed by the error thresholds for D, E and A. In all these examples, increasing *u* leads to A outcompeting D and E. Although both the monopartite and bipartite viruses are adversely affected by deleterious mutations, increasing the mutation rate favors the monopartite virus relative to the bipartite virus.

**Figure 10:**
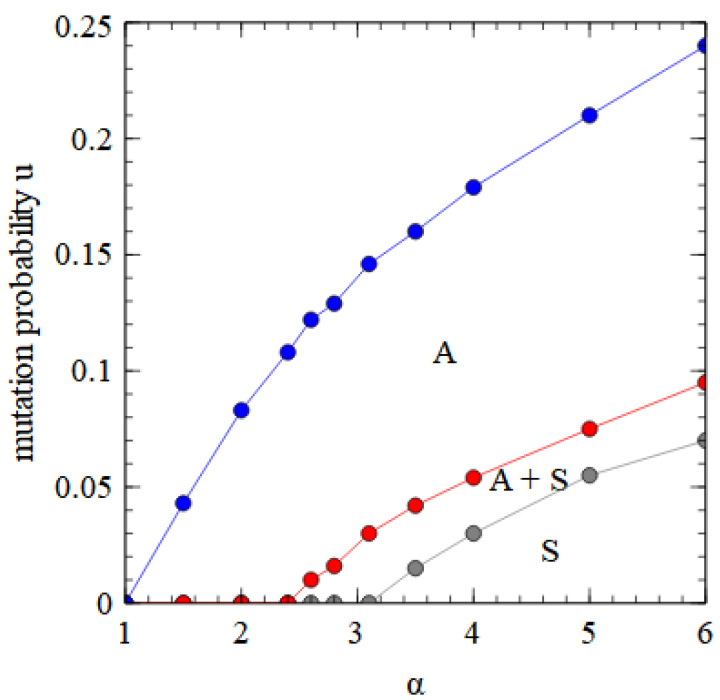
Phase diagram for segmented and monopartite viruses in the assembly model.

We also consider the effect of deleterious mutations in the assembly model. The phase diagram for the fully bipartite case within the assembly model is very similar to that shown in Figure 8 for the strand model. The phase diagram for the fully segmented case in the assembly model is shown in Figure 10. The segmented virus eliminates the monopartite virus at high α and low *u*.

In Figure 8 and Figure 10 we see that a bipartite or a segmented virus can eliminate a monopartite virus at high α and low u. As u increases, there is a transition to a state where D and E are dependent on A, and eventually to a state where only A survives. The fact that bipartite viruses are favored by high transmissibility (or high multiplicity of infection) is not surprising and has been seen in previous theories [25]. However, the fact that bipartite viruses are favored by *low* mutation rates in our models is somewhat surprising, as several previous theories [27,28,29] have argued that bipartite viruses are favored by *high* mutation rates and that the low fidelity of RNA replication in comparison to DNA replication provides an explanation for why bipartite viruses are more common in RNA than DNA viruses. We therefore wished to investigate why our models give qualitatively different results than those obtained from previous theories.

We will discuss our theory in comparison to that of Nee [27]. The theories of [28] and [29] are similar in that bipartite viruses are favored by the low fidelity of RNA replication (however, they differ in other factors). Nee’s theory considers a complete virus C (equivalent to our A) that encodes both a coat protein gene and a polymerase and two types of incomplete sequences I (equivalent to our D and E) that encode either the coat protein or the polymerase. The lengths of C and I sequences are L and L/2. The per-base replication fidelity is q, meaning that the fidelities of replication of C and I are $Q_{C}=q^{L}$ and $Q_{I}=q^{L/2}$. In our model, the fidelities are $Q_{A}={\left(1-u\right)}^{2}$, and $Q_{D}=Q_{E}=(1-u)$. These notations are equivalent if $\left(1-u\right)=q^{L/2}.$ Nee then writes the fitnesses of C and I as $W_{C}=K_{C}q^{L}$ and $W_{I}=K_{I}q^{L/2}R$, where $K_{C}$ and $K_{I}$ are the numbers of copies of a molecule produced per cell if replication occurs, and R is the probability that an incomplete molecule is complemented by co-infection with the other type of strand. He then goes on to say that the bipartite virus should evolve when $W_{I}>W_{C}$. However, we now argue that these formulae for $W_{C}$ and $W_{I}$ are oversimplified for several reasons, and that this appears to lead to incorrect conclusions.

Firstly, Nee’s theory uses single parameters for $K_{C}$ and $K_{I}$. These are similar to the output numbers of viruses per cell in our models; however, we have shown that the numbers of viruses of each type produced per cell depends critically on the other types of viruses that infect the same cell (Table 1 and Table 2 above). The number of A strands produced is reduced substantially when either D or E is in the same cell. The number of D and E strands produced depends on whether D and E are working together as a bipartite virus or whether they are parasites in a cell with A. These factors are essential in our model, but are simply ignored in the theory of Nee [27], and this is one reason why the conclusions of the earlier theory may have been misleading.

Secondly, the replication fidelities discussed above apply to a single round of replication from a functional sequence. However, we have assumed that there are multiple rounds of replication in a single cell. Infection by a single functional A (which we called A_cp_) produces a mixture of functional and mutant sequences: A_cp,_ A_c0_ and A_00_. Although mutant sequences cannot initiate virus replication unless they are complemented by other strands, mutant sequences produced from functional strands can continue to replicate many times in the same cell. We define the effective fidelity of A, $Q_{A}^{eff}$, as the fraction of A_cp_ strands relative to the total number of A produced within a cell, assuming that the infection begins with a functional A_cp_. Similarly, the effective fidelities $Q_{D}^{eff}$ and $Q_{E}^{eff}$ are the fractions of D_c_ and E_p_ relative to the total numbers of D and E, when the infections begin with functional strands.

We considered a case where u=0.1, giving single replication fidelities of $Q_{A}=0.81$, and $Q_{D}=Q_{E}=0.9$. We found that $Q_{A}^{eff}=$ 0.479 when beginning from a single A, and that $Q_{D}^{eff}=0.714,\;Q_{E}^{eff}=0.716$ when beginning from one D and one E. The effective fidelities are much lower than the single replication fidelities. Thus, it cannot be assumed that the fitnesses $W_{C}$ and $W_{I}$ are proportional to the single replication fidelities. This is a second reason why the conclusions of the earlier theory may be unreliable.

Furthermore, the earlier theory does not clearly distinguish between deleterious mutations, which change the sequence without changing the length, and deletions, which reduce the sequence length but leave the remaining sequence intact. In our models, u is the rate of deleterious mutations, not deletions. A mutation in A_cp_ creates A_c0_ or A_0p,_ each of which have the same length as A_cp_. Deletions in A_cp_ might create D_c_ or E_p_, but these have a shorter length and are not equivalent to A_c0_ and A_0p_. We have assumed that D and E were created originally by deletions, but are not produced continually by recurrent deletions, but that mutant A strands are created continually from functional A strands.

We note that the model of Iranzo and Manrubia [25] includes a parameter ρ that they call “loss of segments through mutation and replication fidelity”. Despite its being referred to as mutation, it appears that this parameter represents recurrent deletions, and not deleterious point mutations. Thus, none of the previous models has considered deleterious mutations in the way we have here.

## 4. Discussion

The survey of viral genomes [2] and other references in the introduction show that bipartite and segmented viruses appear to have developed multiple times. However, there are no genera that contain both multipartite and segmented viruses. There are thus no multipartite viruses that are closely related to segmented viruses, suggesting that multipartite and segmented viruses arise separately and that it may not be easy to evolve from one to the other. The models studied here demonstrate likely pathways of evolution from a monopartite virus to either a bipartite or a segmented virus. Our results suggest that there are separate pathways leading to bipartite and segmented viruses that depend on how well short defectives can assemble into separate capsids. Hence, only one or the other of these pathways is likely to emerge from any one monopartite virus.

The pathways to bipartite and segmented viruses both begin with the appearance of complementary defectives. Defective viruses can arise by deletions in a monopartite virus. If the defectives viruses have complementary genes, they can cause the elimination of the monopartite virus. Defective strands have the advantage of being shorter than the complete monopartite virus. Therefore, they will always produce large numbers of RNA strands when in the same cell as the monopartite virus. However, the success of the shorter strands depends also on whether they produce complete virus particles, not only on whether they produce large numbers of RNA strands. The main factor that determines whether evolution proceeds towards a bipartite or a segmented virus is the extent to which short defective strands can be packaged into separate capsids. This depends on whether shorter strands bind capsid proteins in sufficient quantity to nucleate the formation of a virus particle.

In our assembly model, these factors are represented by the rate constant for the assembly step. If the assembly rate constant for the defective particles is equal to that for monopartite virus particles ${(a}_{def}=a_{A}$), then the assembly process works well for defective strands, and defective strands will spread as parasites of the monopartite virus. In this case, we have shown that a variant D*, which starts to associate with E and form S particles, is not selected. In the absence of this association, segmented particles do not form, but D and E can be transmitted separately and, if α is sufficiently high, the bipartite virus D + E can eliminate A. The bipartite combination gains an additional advantage in conditions of excess protein resources, because a larger total number of D and E particles can be produced. The alternative case is when D and E do not assemble efficiently into virus particles ($a_{def}\ll a_{A}$). In this case, we have shown that the variant D* that associates with E is selected by evolution. In this case, S viruses spread and can eliminate the monopartite viruses if α is sufficiently high. We have shown that the segmented virus has an additional advantage under excess RNA conditions.

In our model, we assume that the same number of capsid proteins are used for every virus particle (corresponding to one unit of *PR*). This is be the case if the capsid has a well-defined shape determined by the shape of the capsid proteins. However, if the capsid proteins have the flexibility to assemble into structures of different sizes, then fewer capsid proteins are used to make a capsid enclosing a D or E strand than an A strand. In this case, less than 1 unit of *PR* is used for D and E particles, and it is possible to produce more D and E particles than A particles even in balanced resource conditions. If the possibility of forming smaller capsids for D and E particles exists, this will also make it easier for bipartite viruses to evolve. For capsids that are filamentous or rodlike, the number of capsid proteins required should be proportional to the length of the RNA. This suggests an advantage to bipartite viruses when the capsids are filamentous or rodlike. In fact, several of the examples of origin of bipartite viruses do occur within groups with filamentous or rodlike capsids (Closteroviridae [11], Potyviridae [12], Virgaviridae [13], Ourmiavirus [15]).

We also investigated the effect of deleterious mutations on the competition between virus types. We find that bipartite and segmented viruses both perform better in conditions of low mutation rate (high replication fidelity). Thus, the low fidelity of RNA replication relative to DNA replication cannot be used as an explanation as to why bipartite viruses occur more frequently in RNA than DNA viruses.

## Figures and Tables

**Table 1:** Mean numbers of viruses produced per cell in the strand model. A denotes a complete virus; D is a defective strand encoding a capsid protein gene; and E is a defective strand encoding a polymerase gene.

	Starting Strand Numbers			Mean Numbers of Virus Particles Produced			
	$n_{A}$	$n_{D}$	$n_{E}$	$V_{A}^{out}$	$V_{D}^{out}$	$V_{E}^{out}$	$V_{tot}^{out}$
A	1	0	0	100.0	0.0	0.0	100.0
A + D	1	1	0	30.7	69.3	0.0	100.0
A + E	1	0	1	21.9	0.0	78.1	100.0
A + D + E	1	1	1	14.9	40.9	44.2	100.0
D + E	0	1	1	0.0	47.7	52.3	100.0

**Table 2:** Mean numbers of viruses produced in the Assembly Model in Balanced Resource conditions.

			Starting Strand Numbers			Mean Numbers of Virus Particles Produced				
	$a_{def}$	$r_{S}$	$n_{A}$	$n_{D}$	$n_{E}$	$Z_{A}$	$Z_{D}$	$Z_{E}$	$Z_{S}$	$Z_{tot}$
A	-	-	1	0	0	94.1	0.0	0.0	0.0	94.1
A + D	1	-	1	1	0	30.3	65.3	0.0	0.0	95.7
A + E	1	-	1	0	1	18.6	0.0	77.1	0.0	95.7
A + D + E	1	-	1	1	1	12.8	40.2	44.4	0.0	97.4
D + E fully bipartite	1	-	0	1	1	0.0	46.1	51.1	0.0	97.2
D + E + S mostly bipartite	1	0.01	0	1	1	0.0	36.7	31.8	8.7	77.2
D + E + S	1	0.1	0	1	1	0.0	27.6	6.6	8.3	42.5
D + E + S mostly segmented	0.01	0.01	0	1	1	0.0	22.4	11.1	40.5	74.1
S fully segmented	0	0.01	0	1	1	0.0	0.0	0.0	51.8	51.8
A + S	0	0.01	1	1	1	16.6	0.0	0.0	46.4	63.0

**Table 3:** Numbers of viruses produced by monopartite, bipartite and segmented viruses in three resource conditions.

				Starting Strand Numbers			Mean Numbers of Virus Particles Produced				
		$a_{def}$	$r_{S}$	$n_{A}$	$n_{D}$	$n_{E}$	$Z_{A}$	$Z_{D}$	$Z_{E}$	$Z_{S}$	$Z_{tot}$
A	BR	-	-	1	0	0	94.1	0.0	0.0	0.0	94.1
	XPR	-	-	1	0	0	99.9	0.0	0.0	0.0	99.9
	XRR	-	-	1	0	0	98.0	0.0	0.0	0.0	98.0
D + E	BR	1	0	0	1	1	0.0	46.1	51.1	0.0	97.2
	XPR	1	0	0	1	1	0.0	92.9	103.0	0.0	195.9
	XRR	1	0	0	1	1	0.0	46.7	51.5	0.0	98.2
S	BR	0	0.01	0	1	1	0.0	0.0	0.0	51.8	51.8
	XPR	0	0.01	0	1	1	0.0	0.0	0.0	52.1	52.1
	XRR	0	0.01	0	1	1	0.0	0.0	0.0	68.3	68.3

## Data Availability

Not applicable.
